# DISC1 Regulates Mitochondrial Trafficking in a Miro1-GTP-Dependent Manner

**DOI:** 10.3389/fcell.2020.00449

**Published:** 2020-06-19

**Authors:** Rosalind Norkett, Flavie Lesept, Josef T. Kittler

**Affiliations:** Department of Neuroscience, Physiology and Pharmacology, University College London, London, United Kingdom

**Keywords:** mitochondria, trafficking, schizophrenia, DISC1, miro, GTPase

## Abstract

The disrupted in schizophrenia 1 (DISC1) protein is implicated in major mental illnesses including schizophrenia and bipolar disorder. A key feature of psychiatric disease is aberrant synaptic communication. Correct synaptic transmission is dependent on spatiotemporally regulated energy provision and calcium buffering. This can be achieved by precise distribution of mitochondria throughout the elaborate architecture of the neuron. Central to this process is the calcium sensor and GTPase Miro1, which allows mitochondrial trafficking by molecular motors. While the role of Miro1-calcium binding in mitochondrial transport is well described, far less is known regarding the functions of the two GTPase domains. Here, we investigate the effects of a psychiatric disease-associated mutation in DISC1 on mitochondrial trafficking. We show that this DISC1 mutation impairs Miro1’s ability to transport mitochondria. We also demonstrate the necessity of the first Miro1 GTPase domain in determining direction of mitochondrial transport and the involvement of DISC1 in this process. Finally, we describe the effects of mutant DISC1 on positioning of mitochondria at synapses.

## Introduction

The disrupted in schizophrenia 1 protein (DISC1) has undergone intense study as a candidate susceptibility factor for major mental illness (Brandon and Sawa, 2011; Porteous et al., 2011; Norkett et al., 2017). Disrupted in schizophrenia 1 protein has roles in neuronal proliferation and migration, neuronal development, cytoskeletal dynamics, and intracellular signaling (Kamiya et al., 2006; Kim et al., 2009; Bradshaw et al., 2011; Kvajo et al., 2011; Norkett et al., 2016). Notably, the DISC1 “interactome”—described by an extensive yeast two-hybrid screen (Camargo et al., 2007)—suggests the role of DISC1 as a scaffold protein. The gene was first described due to a large chromosomal rearrangement in a family with a high incidence of psychiatric disorders (Millar et al., 2000) and multiple other DISC1 mutations co-segregate with major mental illness (Thomson et al., 2014).

Among the described interactors of DISC1, proteins involved in microtubule-based transport are highly represented (Camargo et al., 2007; Devine et al., 2016). Disrupted in schizophrenia 1 protein itself adopts a partially mitochondrial localization (James et al., 2004; Park et al., 2010). Previous work from our group and others has shown that DISC1 is a positive regulator of mitochondrial transport (Atkin et al., 2011; Ogawa et al., 2014; Park et al., 2016; Murphy and Millar, 2017) by interacting with the Miro/TRAK mitochondrial trafficking complex and that schizophrenia-associated DISC1 mutations impair mitochondrial transport, function, and fusion (Park et al., 2010; Atkin et al., 2011; Ogawa et al., 2014; Norkett et al., 2016).

Mitochondrial trafficking involves Miro proteins in neurons and other cell types (Saotome et al., 2008; Macaskill et al., 2009b; Russo et al., 2009; Wang and Schwarz, 2009; Lopez-Domenech et al., 2018; Modi et al., 2019). These proteins contain 2 rho GTPase domains, 2 calcium binding EF hand domains, and a transmembrane domain at the C-terminus to anchor them into the outer mitochondrial membrane. With the help of TRAK adaptors, Miro proteins link mitochondria to microtubule motors, allowing their transport and distribution throughout the neuron (Smith et al., 2006; MacAskill et al., 2009a; van Spronsen et al., 2013). Importantly, Miro proteins themselves represent potential signaling hubs. The calcium binding EF hand domains act as a molecular switch for interaction with kinesin motors for precise subcellular localization, e.g., pre- and post-synaptic sites (Macaskill et al., 2009b; Wang and Schwarz, 2009; Stephen et al., 2015; Vaccaro et al., 2017). In *Drosophila*, the activity of the N-terminal GTPase domain of dMiro is necessary for correct mitochondrial distribution in motor neuron axons, and for anterograde mitochondrial flux (Babic et al., 2015). In contrast, far less is known regarding the function of the GTPase domains in mammalian systems.

A disease-associated mutation in the DISC1 gene that has generated considerable interest is that of a 4-base-pair deletion, first described in an American kindred with schizophrenia and schizoaffective disorder (Sachs et al., 2005). This mutation occurs in exon 12 and causes a frameshift, giving rise to a DISC1 transcript lacking 52 amino acids at the C-terminus of the protein. In addition, nine novel amino acids are fused to the truncated DISC1 C-terminus (termed mutDISC1 here). While this mutation has been studied in the context of synaptic communication in induced pluripotent stem cells, the impact of this mutation on mitochondrial dynamics remains unknown (Wen et al., 2014).

Here, we investigate if mutDISC1 can affect mitochondrial trafficking, a key point of healthy neuronal development and synaptic transmission. We show using live imaging in rodent hippocampal cultures that mitochondrial transport is decreased upon expression of this mutant. Further, we determine that the impairment in transport is notable only in the anterograde, kinesin-mediated direction in axons. We also investigate the involvement of Miro1 in this process and show that DISC1 preferentially interacts with a form of Miro that mimics the GTP-bound state (constitutively active version; V13 Miro1). Significantly, we show that V13 Miro1 can rescue the mutDISC1-dependent mitochondrial transport deficit. Finally, by fixed imaging of mitochondria in axons, we demonstrate that mutDISC1 decreases mitochondrial localization to the presynapse, thereby providing a potential mechanism by which this mutation could cause altered synaptic activity, contributing to the onset of psychiatric symptoms.

## Materials and Methods

### Antibodies and Constructs

MtDsRed2, synaptophysin GFP, and WTGFP Miro1 have all been previously described (Fransson et al., 2003, 2006; Macaskill et al., 2009b). GFP V13 and N18 Miro1 were subcloned from myc-tagged constructs (Fransson et al., 2006; MacAskill et al., 2009a) into pEGFP (Clontech). pSuper Miro1 shRNAi with MtDsRed reporter was described in Atkin et al. (2011). Myc-tagged WT Miro1-ires-MtDsRed2 used in Figure 2 has been previously described (Stephen et al., 2015). V13 and N18 versions were generated by subcloning the myc-tagged constructs into this cassette. HA-tagged human DISC1 constructs (WT and mutDISC1) were a kind gift from L. Martin (Pfizer neuroscience). Anti-HA (Haemmagluttinin, 12CA5) and myc (9E10) antibodies were obtained from relevant hybridomas (WB and IF 1:100). Anti-GFP was from Santa Cruz (sc-8334, WB 1:250) or Nacalai Tesque (G090R, IF 1:2000). Anti-human DISC1 (14F2) was previously described (WB and IF 1:100) (Ottis et al., 2011). HRP-conjugated secondaries for Western blot were from Rockland (1:10,000). Fluorescent conjugated secondaries were from Invitrogen (1:1000).

### Cell Culture and Transfection

COS7 cells were maintained and transfected as described in Twelvetrees et al. (2019). Preparation of primary neuronal cultures from E18 pups was performed as previously described (Atkin et al., 2011; Smith et al., 2012, 2014; Norkett et al., 2016). Calcium phosphate precipitation (as in Atkin et al., 2012) or lipofection methods were used for transfection of hippocampal cultures at 7–8 days *in vitro* (DIV) for live imaging or 10 DIV for synaptic occupancy experiments. Lipofection was carried out according to manufacturer’s instructions (Invitrogen) in unsupplemented neurobasal with 6% glucose. Samples were maintained in original conditioned media for 24–48 h for live imaging or 72 h for synaptic occupancy analysis.

### Biochemical Assays and Western Blotting

Co-immunoprecipitation experiments were made in lysis buffer (50 mM Tris, pH 7.5, 0.5% Triton X-100, 150 mM NaCl, 1 mM EDTA, 1 mM PMSF, 1 μg/ml antipain, pepstatin, and leupeptin) using GFP trap beads (Chromotek) on COS cells lysate. SDS–polyacrylamide gel electrophoresis (PAGE) and Western Blotting were carried out as previously described (Norkett et al., 2016). HRP-conjugated secondary antibodies were from Rockland (1:10,000). Bands were visualized using Crescendo Chemiluminescent substrate (Millipore) together with an ImageQuant LAS 4000 CCD camera system (GE Healthcare).

### Immunocytochemistry

Immunocytochemistry and fixed cell imaging were carried out as described in Norkett et al. (2016). Imaging was carried out using a Zeiss LSM 700 upright confocal microscope with a plan Apochromat 63 × oil-immersion lens with 1.4 numerical aperture and ZEN 2010 software. For synaptic occupancy analysis, axonal regions were selected approximately 150 μm from the soma. Images were straightened and thresholded, and image calculator function in ImageJ was used to generate images of colocalized regions between the mitochondrial and synaptic channels.

### Live Cell Imaging

For neuronal imaging of mitochondria, embryonic day 18 (E18) primary hippocampal neurons were transfected at 7–8 DIV and imaged at 9–10 DIV as previously described (Atkin et al., 2012; Norkett et al., 2016). Images were acquired at 1 frame per second for 2 min throughout. Kymographs were created in ImageJ with the straighten and multiple kymograph macros. Resultant kymographs show the process along the *x*-axis and time across the *y*-axis. Motility was assessed by counting the percentage of objects moving during an imaging period. Mitochondria and synaptophysin^*GFP*^-positive vesicles were classed as moving if they moved more than 2 μm between the initial and final frame of acquisition (Macaskill et al., 2009b).

### Statistical Analysis

All data were obtained using cells from three independent preparations unless otherwise stated. Data are presented as mean ± SEM. Individual differences were assessed using Student unpaired *t* test at a 95% significance level. Statistical significance across groups was analyzed using one-way ANOVA with Tukey’s *post hoc* test. NS = not significant, ^∗^*p* < 0.05, ^∗∗^*p* < 0.01, ^∗∗∗^*p* < 0.001.

## Results

### A Schizophrenia-Associated DISC1 Mutation Impairs Anterograde Mitochondrial Trafficking

**FIGURE 1:**
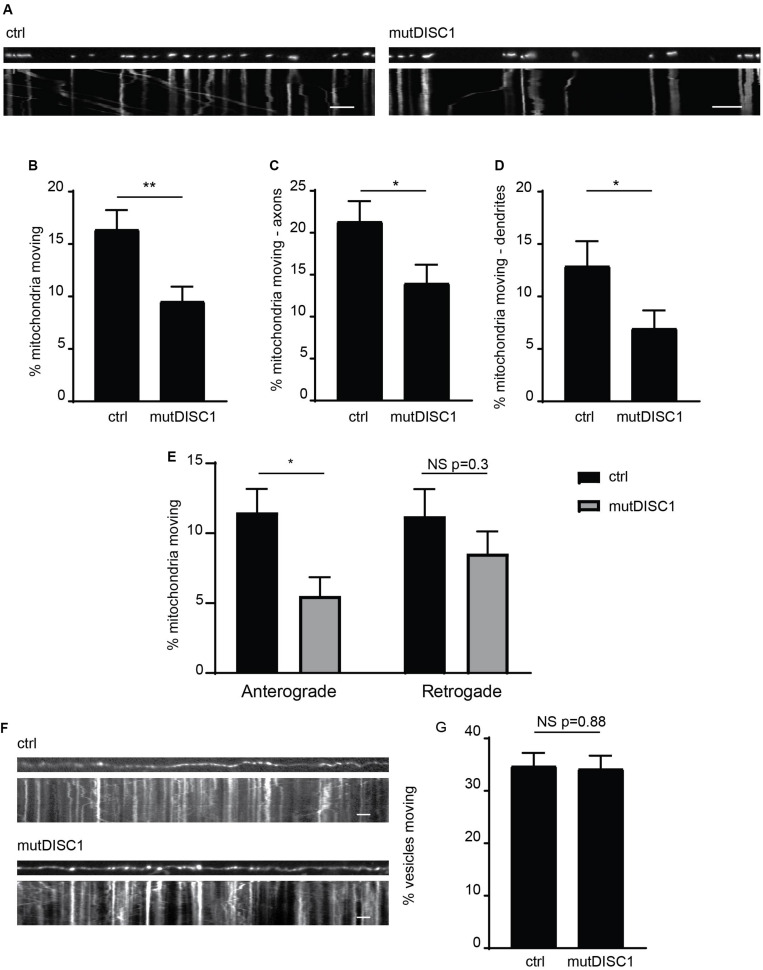
mutDISC1 impairs anterograde mitochondrial transport. **(A)** Kymographs showing impaired mitochondrial trafficking in neuronal axons upon expression of mutDISC1 encoded by the mutDISC1 deletion compared to ctrl (MtDsRed2 alone). **(B)** Quantification of percentage of moving mitochondria in neuronal processes. Expression of mutDISC1 decreases percentages of moving mitochondria (*N* = 34 ctrl and 32 mutDISC1-expressing neurons, *p* = 0.017). Quantification of percentage of moving mitochondria in neuronal axons **(C)**, and dendrites **(D)** showing a decrease upon expression of the mutDISC1 (axons *N* = 14 ctrl and 15 mutDISC1-expressing neurons, *p* = 0.028; dendrites *N* = 20 ctrl and 18 mutDISC1-expressing neurons, *p* = 0.048). **(E)** Analysis of direction of mitochondrial motility assessed as percentage of total mitochondria per axon. mutDISC1 specifically impairs anterograde transport compared to control, while retrograde transport is unaffected (*N* = 14 ctrl and 15 mutDISC1-expressing neurons, anterograde *p* = 0.010, retrograde *p* = 0.298). **(F)** Kymographs showing trafficking of synaptophysin GFP-positive vesicles upon expression of mutDISC1. **(G)** Quantification of synaptic vesicle trafficking in neuronal axons shows no effect of mutDISC1 compared to control (*N* = 16 ctrl and 19 mutDISC1-expressing neurons, NS *p* = 0.877).

We and others have previously identified that DISC1 acts as a positive regulator of mitochondrial trafficking, with varying inhibitory effects of schizophrenia-associated mutations reported (Atkin et al., 2011; Ogawa et al., 2014; Norkett et al., 2016; Park et al., 2016). One mutation, which has yet to be investigated in such a way, is the mutant DISC1 protein generated from a 4-bp deletion in the DISC1 gene that gives rise to a frameshift mutation (Supplementary Figure 1A; Sachs et al., 2005; Wen et al., 2014). This mutDISC1 can still be recruited to mitochondria by Miro1 (Supplementary Figure 1B) as we have previously shown for WT DISC1 (Norkett et al., 2016). To investigate any effects of this mutation on mitochondrial trafficking, we expressed MtDsRed2 (labeling mitochondria) in cultured primary neurons at DIV 7 either alone (ctrl) or together with mutDISC1 and carried out live imaging at DIV 9–10. DISC1 expression was confirmed by *post hoc* immunostaining for HA. Analysis of moving mitochondria revealed a 36% decrease in mitochondrial motility upon expression of mutDISC1 (Figures 1A,B. 16.4 ± 1.8% of mitochondria were moving in control, vs 9.6 ± 1.4% in mutDISC1-expressing cells, *p* = 0.004).

Next, we investigated the effect of this DISC1 mutation on mitochondrial trafficking in axons and dendrites, as we and others have previously shown that DISC1 can upregulate trafficking in both of these compartments (Atkin et al., 2011; Ogawa et al., 2014; Norkett et al., 2016; Park et al., 2016). Instead, we found that mutDISC1 caused a decrease in trafficking in both compartments (Figures 1C,D, dendrites 13.0 ± 2.3% of mitochondria were moving in control, vs 7.0 ± 1.7% in mutDISC1-expressing cells, *p* = 0.049, Ctrl axons 21.4 ± 2.4%, mutDISC1 axons 14.1 ± 2.1%). Unlike dendritic microtubules, axonal microtubules have uniform polarity, with the plus ends facing away from the soma (Kapitein and Hoogenraad, 2015). This allowed us to determine if kinesin-mediated (plus-end directed) or dynein-mediated (minus-end directed) transport is affected by mutDISC1. Interestingly, we found that mutDISC1 expression led to a 50% decrease in anterograde, kinesin-mediated trafficking, whereas retrograde, dynein-mediated transport was unaffected (Figure 1E Anterograde ctrl = 11.5 ± 1.7%, mutDISC1 = 5.5 ± 1.3%, *p* = 0.010, retrograde ctrl = 11.2 ± 2.0, mutDISC1 = 8.5 ± 1.6%, *p* = 0.298). This was not due to a general effect on microtubule transport, as mutDISC1 expression had no effect on transport of synaptophysin^*GFP*^-positive vesicles (Figure 1F, quantified in Figure 1G, ctrl = 34.8 ± 2.4% vesicles motile, mutDISC1 = 34.3 ± 2.4% vesicles motile, NS *p* = 0.877). Therefore, mutDISC1 does not disrupt all microtubule-based transport, but instead affects specific cargoes, including mitochondria—consistent with our previous findings (Norkett et al., 2016). This is also consistent with a previous report suggesting that kinesin-dependent mitochondrial trafficking may be more affected by DISC1 than dynein-based transport (Ogawa et al., 2014).

### The GTPase State of Miro1 GTPase Domain I Impacts Anterograde Mitochondrial Transport and DISC1 Binding

**FIGURE 2:**
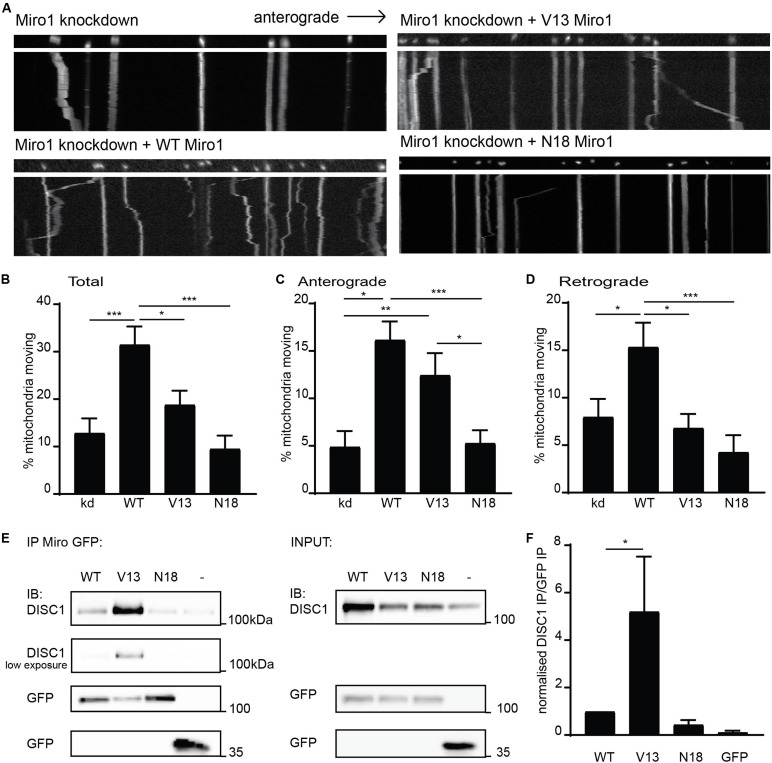
Miro1 GTPase domain I state influences anterograde mitochondrial trafficking and DISC1 interaction. **(A)** Kymographs showing knockdown rescue experiments of mitochondrial trafficking upon knockdown of Miro1 and rescue with WT, V13, and N18 Miro1 in neuronal axons. **(B)** Quantification of mitochondrial trafficking reveals that expression of WT Miro1 can rescue mitochondrial trafficking after knockdown, whereas expression of GTPase domain I mutants V13 and N18 Miro1 cannot rescue the defect (*N* = 18 cells for all conditions, kd vs WT *p* < 0.001, kd vs V13 NS, kd vs N18 NS, WT vs V13 *p* < 0.05, WT vs N18 *p* < 0.001). **(C)** Quantification of anterograde mitochondrial transport as percentage of total mitochondria per axon shows that WT and V13 Miro1 can rescue mitochondrial trafficking, while N18 cannot (*N* = 18 cells for all conditions, kd vs WT *p* < 0.001, kd vs V13 *p* < 0.01, kd vs N18 NS, WT vs V13 NS, WT vs N18 *p* < 0.001, V13 vs N18 *p* < 0.05). **(D)** Quantification of retrograde mitochondrial transport shows that WT can rescue mitochondrial trafficking while V13 and N18 cannot (*N* = 18 cells for all conditions, kd vs WT *p* < 0.05, kd vs V13 NS, kd vs N18 NS, WT vs V13 *p* < 0.05, WT vs N18 *p* < 0.001). **(E)** GFP trap experiment from COS7 cells showing coIP of DISC1 with GFP-tagged Miro1 WT, V13, or N18. **(F)** Quantification of co-immunoprecipitated DISC1 band normalized to immunoprecipitated GFP Miro band. There is an increased amount of DISC1 pulled down with constitutively active, V13 Miro 1 compared to WT Miro1 (*N* = 5, *p* = 0.05).

To further investigate mechanisms specifying direction of mitochondrial transport, we focused on Miro1, a crucial mitochondrial trafficking adaptor and DISC1 interactor. To address this, we used shRNA to knockdown Miro1 in hippocampal cultures and assessed mitochondrial trafficking in axons upon re-introduction of WT Miro1, V13 Miro1 (mimicking a GTP-bound version of the domain), or N18 Miro1 (mimicking a GDP-bound version of the domain) (Fransson et al., 2003). We found that WT Miro1 was able to rescue trafficking of MtDsRed2-labeled mitochondria compared to knockdown, increasing trafficking by 190% (Figures 2A,B, knockdown = 10.8 ± 2.3% mitochondria moving, WT = 31.6 ± 3.4% mitochondria moving, *p* < 0.001). While V13 Miro1 was able to exert a small but non-significant effect, N18 Miro1 was not able to rescue mitochondrial trafficking compared to knockdown (V13 = 19.0 ± 3.0% mitochondria moving NS compared to knockdown, N18 = 9.6 ± 2.7% mitochondria moving NS compared to knockdown). Interestingly, when moving mitochondria were split into anterogradely and retrogradely moving, we found that the V13 behaved differently to WT and N18 Miro1. WT Miro1 was able to rescue mitochondrial trafficking in both directions (anterograde; knockdown = 4.9 ± 1.6%, WT = 16.2 ± 1.9%, *p* < 0.001 retrograde; knockdown = 8.0 ± 1.9%, WT = 15.4 ± 2.5% *p* < 0.05). In contrast, we found that the V13 mutant was only able to rescue anterograde mitochondrial transport comparably to WT (V13 = 12.4 ± 2.3%, *p* < 0.01, Figure 2C, retrograde trafficking V13 = 6.8 ± 1.5% not significant compared to knockdown, Figure 2D), while the dominant negative, N18 Miro1, was not able to rescue transport in either direction compared to knockdown (N18 = 5.3 ± 1.3%, Figure 2C, N18 = 4.3 ± 1.8% not significant compared to knockdown, Figure 2D). Thus, anterograde mitochondrial transport is dependent on the GTPase state of Miro1, with the dominant negative form preventing trafficking, and the constitutively active form promoting anterograde transport specifically.

We have previously shown that the interaction between DISC1 and Miro1 contributes to mitochondrial transport (Norkett et al., 2016). The parallels between loss of anterograde trafficking with a DISC1 mutation and with N18 Miro1 prompted the question as to whether these factors may act in a common pathway—i.e., DISC1 and V13 Miro1 act in concert to promote kinesin mediated trafficking. Therefore, we investigated the possibility that the state of this Miro domain might alter the DISC1–Miro1 interaction, and so, exert an increase in mitochondrial transport. We carried out GFP trap coimmunoprecipitation experiments from COS7 cells expressing untagged, WTDISC1, and GFP-tagged WT, V13, or N18 Miro1 to mimic GTP- and GDP-bound states, respectively. Interestingly, Western blot analysis revealed that more DISC1 is immunoprecipitated with V13 Miro1 compared to WT Miro1 (Figure 2E, quantified in 2F, normalized DISC1 intensity for WT Miro1 = 1.0, for V13 Miro1 = 5.2 ± 2.1, *p* = 0.05), indicating that DISC1 preferentially interacts with the GTP-bound Miro mimic. We also confirmed that mutDISC1 can be recruited to mitochondria in a Miro-dependent manner (Supplementary Figure 1B). Notably, WT and V13 Miro (but not N18 Miro) can recruit mutDISC1 to mitochondria in neurons. Significantly, this enhanced interaction may explain the reason for Miro1 V13’s capability of promoting anterograde mitochondrial transport as DISC1 upregulates kinesin-based motility (Ogawa et al., 2014), as also suggested by loss of anterograde trafficking with mutDISC1.

### Constitutively Active Miro1, but Not Dominant Negative Miro1, Can Rescue the Mutant DISC1-Induced Trafficking Defect

**FIGURE 3:**
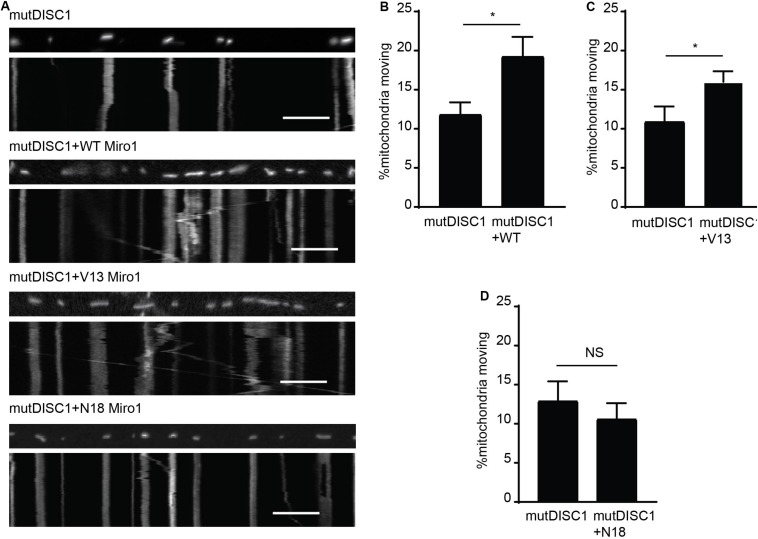
Miro1 can rescue mutDISC1 mitochondrial trafficking defect in a GTPase dependent manner. **(A)** Kymographs showing effect of expressing Miro1 WT, V13, or N18 in conjunction with mutDISC1. WT Miro1 **(B)** and V13 Miro1 **(C)** increase mitochondrial transport compared to control (mutDISC1only) whereas N18 Miro1 does not **(D)** (*N* = 15 mutDISC1-expressing neurons and 26 mutDISC1 + WT Miro-expressing neurons, *p* = 0.036, *N* = 16 mutDISC1-expressing neurons and 24 mutDISC1 + V13 Miro-expressing neurons, *p* = 0.033, *N* = 10 mutDISC1-expressing neurons and 19 mutDISC1 + N18 Miro-expressing neurons, *p* = 0.483).

That DISC1 and V13 Miro1 may specifically cooperate to mediate anterograde mitochondrial trafficking warranted further investigation. Thus, we carried out mitochondrial trafficking assays with co-expression of WT, V13, or N18 Miro1 and mutDISC1 to discern any possible rescue effect by Miro. We confirmed equal protein expression levels of each of the Miro isoforms by immunocytochemistry (Supplementary Figure 1C). We found that Miro1 could indeed rescue the trafficking defect, and that this was dependent on the state of GTPase domain I. Figure 3A shows example kymographs of mutDISC1 overexpression compared to addition of WT, V13, or N18 Miro1. Percentage of moving mitochondria in each condition is quantified in Figures 3B–D. Upon co-expression of WT Miro1 with mutDISC1, percentage of mitochondria moving increased around 60% (mutDISC1 = 11.9 ± 1.5%, mutDISC1 + WT = 19.3 ± 2.4%, *p* = 0.036). Likewise, expression of Miro1 V13 increased trafficking by around 45% (mutDISC1 = 11.0 ± 1.9%, mutDISC1 + V13 = 16.0 ± 1.4%, *p* = 0.033). However, N18 Miro 1 was unable to affect any change in mitochondrial transport in comparison to mutDISC1 (mutDISC1 = 13.0 ± 2.4%, mutDISC1 + N18 = 10.7 ± 1.6%, *p* = 0.483).

### The mutDISC1 Decreases Mitochondrial Synaptic Occupancy

**FIGURE 4:**
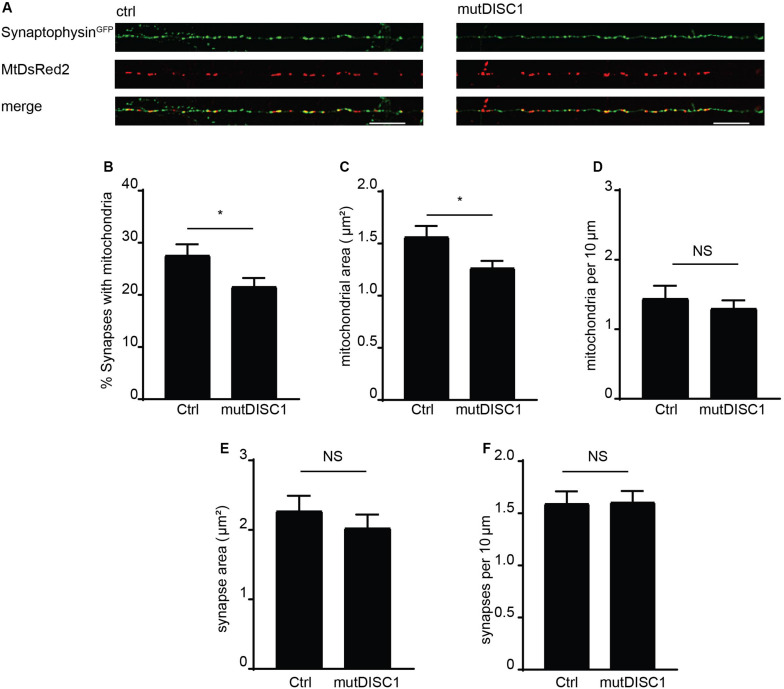
mutDISC1 impairs positioning of mitochondria at the presynapse. **(A)** Confocal images showing colocalization of mitochondria with presynaptic sites labeled with synaptophysin GFP (control) or upon expression of the mutDISC1. **(B)** Percentage of synapses colocalizing with mitochondria is decreased upon expression of mutDISC1 (*N* = 11 ctrl and 11 mutDISC1-expressing neurons, *p* = 0.028). **(C)** Mitochondrial area is decreased upon expression of DISC1 4 bp deletion (*p* = 0.017). **(D)** Mitochondrial density is unaffected by expression of mutDISC1 (*p* = 0.508). Synaptic area **(E)** and density **(F)** are unchanged by expression of mutDISC1 (*p* = 0.933 and *p* = 0.392, respectively).

Mitochondrial trafficking is of exceptional importance within the neuron due to specialized sites of high energy and calcium buffering demand (Birsa et al., 2013). One such example is the presynapse where calcium rises trigger vesicular release of neurotransmitter (Borst and Sakmann, 1996; Schneggenburger and Neher, 2000) and so must be buffered to attenuate this signal (Kwon et al., 2016; Vaccaro et al., 2017; Devine and Kittler, 2018). Furthermore, the mutDISC1 has been previously shown to cause a defect in presynaptic release and frequency facilitation (Kvajo et al., 2011; Wen et al., 2014). Thus, we examined the prospect that disrupted mitochondrial trafficking—due to mutDISC1—might alter positioning of mitochondria at the presynapse. To address this, fixed confocal imaging was carried out on DIV 13 neurons expressing MtDsRed2 and synaptophysin^*GFP*^ to label mitochondria and presynaptic specializations, respectively (Figure 4A). Colocalization analysis revealed that around 28% of synapses colocalize with mitochondria under control conditions. However, this value was decreased by around 20% upon expression of mutDISC1 (Figure 4B ctrl = 27.7 ± 1.9%, mutDISC1 = 21.7 ± 1.5%, *p* = 0.028). We also found that mutDISC1 expression led to a 20% reduction in mitochondrial localization to synapses (from 50% in control conditions to 40% with mutDISC1 expression, data not shown).

In addition to position of mitochondria in relation to synapses, we also analyzed their size (Figures 4C,E). While the area of presynaptic terminals was unchanged (ctrl = 1.60 ± 0.11 μm^2^, mutDISC1 = 1.61 ± 0.11 μm^2^, NS, *p* = 0.93), the mitochondrial area was decreased by 19% upon expression of mutDISC1 (ctrl = 1.57 ± 0.10 μm^2^, mutDISC1 = 1.27 ± 0.06 μm^2^, *p* = 0.017). This decrease in area of mitochondria is consistent with our previous data where we show another DISC1 mutation decreases mitochondrial fusion, likely due to the interaction between DISC1 and mitochondrial fusion proteins, mitofusins (Norkett et al., 2016). Further, these smaller mitochondria may be less functional than in control conditions, as mitochondrial morphology and function are tightly linked (Westermann, 2010). This would be consistent with reports of DISC1 being important for maintaining mitochondrial membrane potential and expression of mutDISC1 disrupting this process (Park et al., 2010; Eykelenboom et al., 2012).

In order to rule out the possibility that the diminished colocalization was caused by a decrease in number of synapses or mitochondria in the imaged regions of axons, we also calculated their density (Figures 4D,F). We found no significant alteration in numbers of mitochondria per 10 μm of axon (ctrl = 1.45 ± 0.18, mutDISC1 = 1.31 ± 0.11 mitochondria per 10 μm, NS, *p* = 0.51). Nor did we detect any difference in numbers of synaptophysin GFP puncta (ctrl = 2.28 ± 0.21, mutDISC1 = 2.03 ± 0.19 puncta per 10 μm, NS, *p* = 0.39). Therefore, the decreased colocalization of mitochondria with presynaptic boutons is not due to alteration in numbers of either, rather in the positioning of mitochondria.

Taken together, these data suggest this DISC1 mutation prevents anterograde mitochondrial transport and so causes an improper distribution of mitochondria throughout the axon. Therefore, there are fewer presynaptic sites with mitochondria present. This anterograde transport defect can be overcome with V13 Miro1, mimicking the GTP-bound state of Miro1—the form with which DISC1 preferentially interacts.

## Discussion

Here, we demonstrate that the mutDISC1 encoded by the schizophrenia-associated 4-bp deletion in DISC1 impairs anterograde mitochondrial transport in neuronal axons. Further, we reveal a role of the first GTPase domain of Miro1 on mitochondrial transport in mammalian neurons. We find that the constitutively active form of Miro1 can specifically rescue anterograde mitochondrial transport upon Miro1 knockdown, while the dominant negative form does not. Moreover, V13 Miro1 rescues the altered mitochondrial trafficking caused by expression mutDISC1 and we show the Miro1 GTPase state influences the biochemical interaction with DISC1. Finally, we show that the mutDISC1 causes a decrease in mitochondria at the presynapse.

Disrupted in schizophrenia 1 protein is proposed to positively modulate mitochondrial trafficking via biochemical association with Miro and TRAK proteins—themselves positive regulators of mitochondrial transport—as well as interaction with syntaphilin, an anchor protein (Atkin et al., 2011; Ogawa et al., 2014; Norkett et al., 2016; Park et al., 2016). We found that this schizophrenia-associated deletion in DISC1 drastically reduces mitochondrial transport in hippocampal axons and dendrites. Multiple DISC1 mutants have adverse effects on mitochondrial transport (Atkin et al., 2011; Ogawa et al., 2014; Norkett et al., 2016). Expression of a fusion protein of DISC1 and Boymaw [also called DISC1FP1 (Zhou et al., 2008; Eykelenboom et al., 2012), a potential outcome of a schizophrenia-associated chromosomal translocation] drastically reduces mitochondrial transport and also mitochondrial fusion (Norkett et al., 2016). In our mutDISC1 experiments, it is interesting to note this specific decrease in anterograde transport, which is consistent with the described effect of the R37W DISC1 mutation (Ogawa et al., 2014).

Importantly, we also report that the GTPase state of Miro1 influences the direction of mitochondrial transport in agreement with results from a *Drosophila* model (Babic et al., 2015). Knocking out Miro1 in *Drosophila* leads to a drastic decrease in mitochondrial trafficking that can be rescued in the anterograde direction by a constitutively active version. However, a dominant negative—mimicking the GDP-bound state—was unable to rescue this effect. Crucially, our findings extend data showing the importance of GTP-bound Miro1 for anterograde transport in mammalian systems. The GTPase activity of Miro1’s N-terminus has been demonstrated *in vitro* (Lee et al., 2016). These data imply a necessity for Miro1 in anterograde transport, which cannot be compensated by Miro2 (also present in our mammalian system). This is consistent with our previous findings that Miro1 is the major isoform responsible for mitochondrial trafficking in neurons (Lopez-Domenech et al., 2016).

Notably, we propose that this anterograde transport may be facilitated by DISC1. By coimmunoprecipitation, DISC1 preferentially interacts with the GTP-bound mimic form of Miro. While this is the first GTPase-dependent interaction confirmed biochemically, the GTPase state of Miro has also been shown to influence recruitment of a centromere associated protein—CENP-F—to mitochondria, whereas the GDP-bound mimic does not (Kanfer et al., 2015). Moreover, our data show that V13 Miro1 can rescue the mutDISC1 phenotype of impaired mitochondrial transport, while the N18 Miro1 cannot. This suggests a role for DISC1 in influencing the GTPase state of Miro. For example, DISC1 may act as a scaffold to recruit Miro1 GTPase-activating proteins (GAPs) or guanidine exchange factors (GEFs) to the mitochondrial trafficking complex. In this way, DISC1 could locally influence moving mitochondria’s direction via Miro1’s GTPase state and, potentially, the dominant molecular motor. The GTP-bound form for Miro1 could favor kinesin-based transport, causing the bias toward anterograde trafficking. The mutDISC1 may confer a loss of function in this regard, which can be overcome by expression of V13 but not N18 Miro1. It would be interesting to investigate Miro-GTP levels in the presence of WT DISC1 or mutDISC1 in live cells. Coupling a GTP/GDP sensor (as described in Bianchi-Smiraglia et al., 2017) to Miro would allow live detection of the bound nucleotide and could even be correlated with the direction of transport—e.g., are anterogradely moving mitochondria likely to be labeled with Miro-GTP? The effect of perturbing DISC1 expression could thus be readily studied.

The regulation of the GTPase state of Miro, and its cellular effects, are of great interest for further study. A GEF for Miro has been proposed in *Drosophila* and has conserved roles in a mammalian system. The Vimar protein (RAP1GDS1 in mammals) encodes an atypical GEF and is involved in mitochondrial fission and fusion via Miro. Vimar RNAi also decreases mitochondrial transport, consistent with the importance of the GTPase state of Miro in mitochondrial transport (Ding et al., 2016). Beyond GAPs and GEFs, the kinase Polo has been shown to phosphorylate *Drosophila* Miro in its N-terminal GTPase domain (Lee et al., 2016). This phosphorylation enhances the hydrolyzing activity of this GTPase domain. Therefore, it would be important to elucidate the effect of this kinase on Miro-dependent mitochondrial transport as well as identify further regulators of Miro’s GTPase activity.

At the cellular level, we show that mutDISC1 interrupts normal mitochondrial localization, decreasing the number of presynapses with mitochondria present. Correct mitochondrial distribution is of particular importance within neurons because sustaining neuronal excitability and synaptic transmission requires a lot of energy (Birsa et al., 2013; Sheng, 2017; Devine and Kittler, 2018). This mislocalization of mitochondria at the presynapse may modulate presynaptic calcium signals and disrupt ATP provision and, thus, neuronal transmission (Kwon et al., 2016; Vaccaro et al., 2017; Devine and Kittler, 2018). Indeed, mutDISC1 has been previously linked to impaired presynaptic activity. In glutamatergic neurons derived from induced pluripotent stem cells from a patient with the 4-bp mutation, there was a decrease in synaptic transmission (Wen et al., 2014). Mutations in DISC1 have also been linked to altered axonal targeting (Kvajo et al., 2011) and shorter, less branched dendritic arbors (Lepagnol-Bestel et al., 2013), while impairing DISC1-dependent mitochondrial trafficking disrupts dendritic morphogenesis (Norkett et al., 2016).

To conclude, our results further support an important role for DISC1 as a regulator of mitochondrial transport. It remains to be determined how mutDISC1 may affect neuronal morphology, and if these phenotypes are dependent on atypical mitochondrial distribution.

## Data Availability Statement

The datasets generated for this study are available on request to the corresponding author.

## Ethics Statement

All experimental procedures were carried out in accordance with institutional animal welfare guidelines and licensed by the UK Home Office in accordance with the Animals (Scientific Procedures) Act 2013.

## Author Contributions

RN and JK: conceptualization. RN, FL, and JK: methodology and writing—review and editing. RN and FL: formal analysis and investigation.

## Conflict of Interest

The authors declare that the research was conducted in the absence of any commercial or financial relationships that could be construed as a potential conflict of interest.

## Supplementary Material

FIGURE S1 The DISC1 4 base pair deletion. **(A)** Schematic showing schizophrenia associated DISC1 4 base pair deletion and equivalent expression construct mutDISC1 encoded by the mutation. **(B)** mutDISC1 is recruited to mitochondria in a Miro-dependent manner in DIV9-10 hippocampal neurons Scale bar 10 μm. Line scans show MtsDsRed and mutDISC1 intensity in the boxed region. The distribution of mutDISC1 changes from cytosolic and nuclear to mitochondrial as shown by colocalization the MtDsRed2. **(C)** Quantification of Miro isoform expression by immunocytochemistry and fluorescence intensity shows comparable expression levels.
