# Supplementary figures and images for: DISC1 Regulates Mitochondrial Trafficking in a Miro1-GTP-Dependent Manner
Source: Front Cell Dev Biol. 2020 Jun 19;8:449. doi: 10.3389/fcell.2020.00449 (PMC7317294; doi:10.3389/fcell.2020.00449)

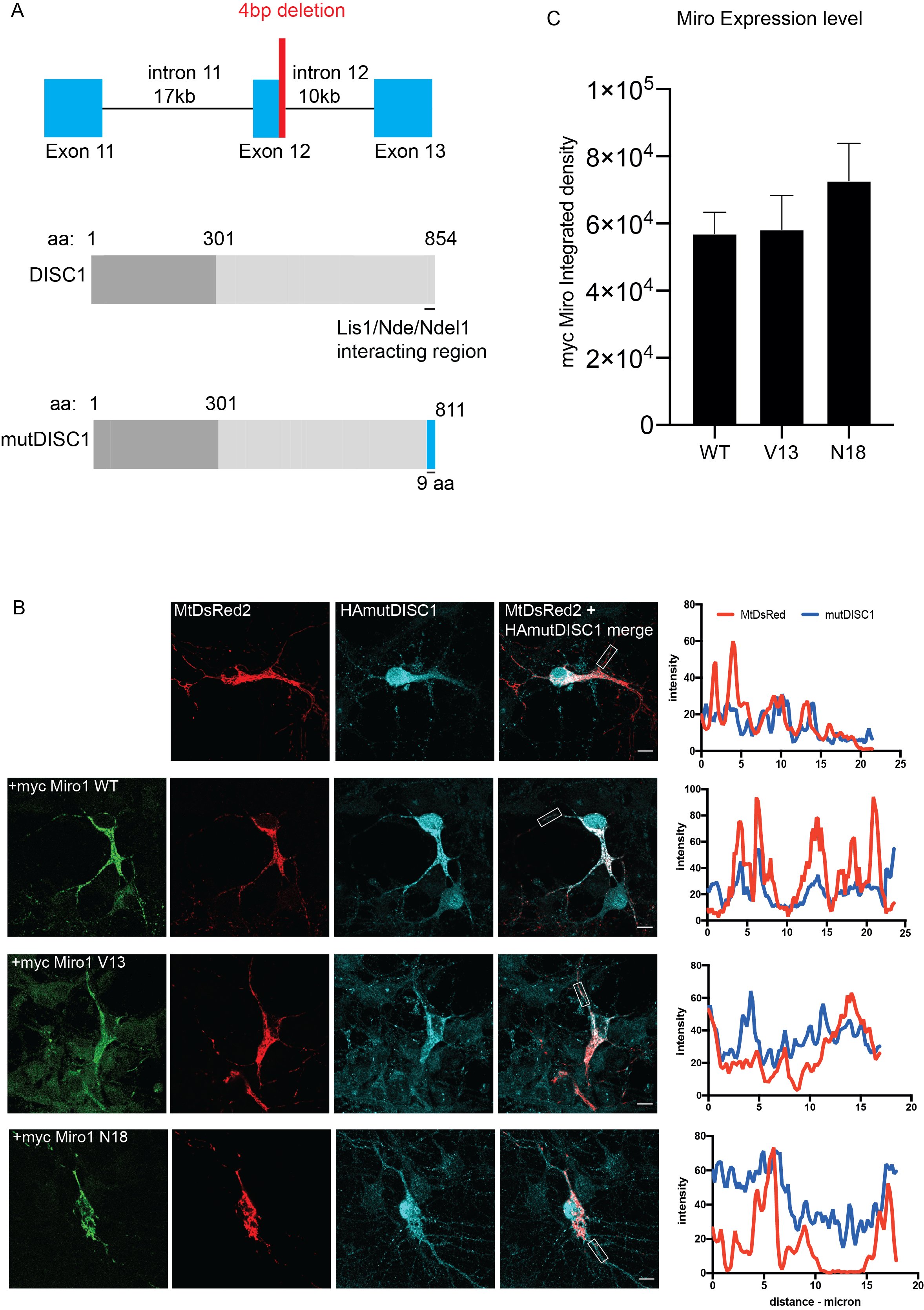

Supplement: FIGURE S1 — The DISC1 4 base pair deletion. (A) Schematic showing schizophrenia associated DISC1 4 base pair deletion and equivalent expression construct mutDISC1 encoded by the mutation. (B) mutDISC1 is recruited to mitochondria in a Miro-dependent manner in DIV9-10 hippocampal neurons Scale bar 10 μm. Line scans show MtsDsRed and mutDISC1 intensity in the boxed region. The distribution of mutDISC1 changes from cytosolic and nuclear to mitochondrial as shown by colocalization the MtDsRed2. (C) Quantification of Miro isoform expression by immunocytochemistry and fluorescence intensity shows comparable expression levels. [file Image_1.JPEG]
